# Vitamin D Supplementation in Overweight/obese Asian Indian Women with Prediabetes Reduces Glycemic Measures and Truncal Subcutaneous Fat: A 78 Weeks Randomized Placebo-Controlled Trial (*PREVENT-WIN* Trial)

**DOI:** 10.1038/s41598-019-56904-y

**Published:** 2020-01-14

**Authors:** Surya Prakash Bhatt, Anoop Misra, Ravindra Mohan Pandey, Ashish Datt Upadhyay, Seema Gulati, Namrata Singh

**Affiliations:** 1 Diabetes Foundation (India), Safdarjung Development Area, New Delhi, 110016 India; 2 National Diabetes Obesity and Cholesterol Foundation (N-DOC), Safdarjung Development Area, New Delhi, 110016 India; 3 Department of Pulmonary Medicine and Sleep Disorders, All India Institute of Medical Sciences, Ansari Nagar, New Delhi, 110029 India; 4 Biostatistics, All India Institute of Medical Sciences, Ansari Nagar, New Delhi, 110029 India; 5 Fortis C-DOC Center of Excellence for Diabetes, Metabolic Diseases, and Endocrinology, B-16, Chirag Enclave, New Delhi, India

**Keywords:** Pre-diabetes, Pre-diabetes

## Abstract

Vitamin D deficiency may contribute to etiology of type 2 diabetes in Asian Indians. The objectives of this study was to evaluate effect of vitamin D supplementation on glycemic profile and body composition in prediabetic and vitamin D deficient overweight/obese Asian Indian women. In this open-label randomized placebo-controlled trial (78 weeks duration), 121 females (aged 20–60 years) with prediabetes and vitamin D deficiency were randomly allocated in intervention (n, 61) and placebo (n, 60) groups. The primary outcome variables were fasting blood glucose (FBG), 2-h blood glucose post OGTT (2-h BG), hemoglobin A1c (HbA1C), and reversal to normoglycemia. In Intention-to-treat analysis, at the end of intervention, we observed significant decrease in FBG [−5.0 (−12.6–2.4), p = 0.04], 2-h blood glucose post OGTT [−11(−49.3–26.9), p = 0.02], hemoglobin A1c [−0.41 (5.89, 6.55), p = 0.05] and increase in 25(OH) D [7.5 (−6.0–20.9), p = 0.002] levels in intervention as compared to the placebo group. Changes in glycemic category based on FBG were as follows; intervention group: normal FBG, 58.6%; impaired fasting glucose (IFG), 39%; and type 2 diabetes mellitus (T2DM), 2.4%; placebo group: normal FBG, 48.8%; IFG, 46.3%; and T2DM, 4.9%. Changes in category of 2-hour glucose post OGTT after intervention were as follows; intervention group: normal glucose tolerance (NGT) 51.2% and prediabetes, 48.8%; placebo group: NGT, 43.9%; prediabetes, 53.7% and T2DM, 2.4%. After intervention, subscapular skinfold (visit I^st^ compared to visit III^rd^) and suprailiac skinfold (visit II^nd^ compared to visit III^rd^) were significantly lower in intervention group *vs*. control group. In conclusion, we observed significant reduction in FBG, 2-hour glucose post OGTT, HbA1c, and truncal subcutaneous fat and reversal to normoglycemia in overweight/obese prediabetic vitamin D deficient Asian Indian women after 78 weeks of vitamin D supplementation.

## Introduction

Vitamin D deficiency is increasingly recognised as a global health problem. Data from Indian subcontinent show high prevalence of vitamin D deficiency. Although not much comparative data are available, one study shows lower plasma 25 hydroxy vitamin D (25(OH)D) levels in South Asians than White caucasians^1^. Specifically, in India, prevalence of vitamin D deficiency is high (94%) in urban areas primarily because of poor exposure to sunlight on account of urbanization, mechanization and office-based jobs^2^. Our recent data show that vitamin D deficiency is common even in rural areas of India despite high degree of sun exposure^3^. In Asian Indians residing in India, we have previously s hown associations of serum 25(OH) D levels with abdominal obesity^4^ and non-alcoholic fatty liver disease^5^.

Prediabetes is an intermediate state of hyperglycemia and gives an opportunity to intervene so that this state could be reverted to normal glucose regulation. Asian Indians have one of the highest number of individuals with pre-diabetes and diabetes among all major ethnic groups^6^, and the conversion from prediabetes to diabetes occurs more rapidly in this population than other ethnic groups^7,8^. Dietary and lifestyle approaches have been tried to prevent progression of diabetes from prediabetes with some success in other countries, and in India^9^. However, because of higher risk of conversion from prediabetes to diabetes in Asian Indians, more aggressive and innovative approaches are needed.

Worldwide, there are several studies which connect vitamin D deficiency with insulin resistance, metabolic syndrome and diabetes. In a cross-sectional study of 488 subjects, those having prediabetes and low circulating 25(OH)D concentration were most insulin resistant and had impaired beta cell function^10^. Those with low vitamin D levels also showed highest progression to diabetes over 8–10 year period in Swedish population^11^. Such relationships of vitamin D deficiency and hyperglycemia has been shown in populations other than Whites as well; in Middle Eastern (Kuwait)^12^, Korean^13^, and Asian Indian residing in India^14,15^. Specifically, in 157 Asian Indians residing in western India, vitamin D deficiency/insufficiency was found in 115 (73.25%) individuals with prediabetes and those with lowest 25(OH) D levels (<10 ng/ml) had the highest insulin resistance^14^.

While general population has been subjected to research on Vitamin D deficiency in most studies, investigators have not specifically focussed on women, although higher odds of vitamin D deficiency in women has been reported from India^16^. Further, it has been shown that Asian Indians have greater vitamin D deficiency than other ethnic groups^17^. Asian Indian women may be more predisposed to develop vitamin D deficiency than whites in developed countries due to large body area coverage with traditional clothes and some reservations to outbound activities because of social and other reasons. In recently conducted study exclusively on women in New Delhi and adjoining rural areas, we have reported marked prevalence of vitamin D deficiency (94.5%; 68.6% deficiency and 25.9% insufficiency in urban and 90.8% in rural areas)^3,15^. In former study, which was conducted in women with prediabetes residing in urban New Delhi, lower vitamin D levels are associated with higher fasting blood glucose values (FBG)^15^. In this paper, we emphasized that, in view of this association, vitamin D intervention studies are needed in Asian Indian women with prediabetes to evaluate if diabetes could be prevented.

We hypothesized that, in Asian Indian women with prediabetes, supplementation of vitamin D could lead to decrease in glycemia and reversal of prediabetes to normal glucose regulation, along with improvement in insulin sensitivity and body composition. In the current study, we aimed to determine the role of vitamin D supplementation on progression of prediabetes to diabetes and reversal to normoglycemia, effects on insulin resistance, body composition, and metabolic profile among overweight/obese adult women with the prediabetes and vitamin D deficiency.

## Methods

The study [*Prevent*ion of type 2 diabetes (T2DM) in *w*omen with prediabetes using vitamin D supplementation and lifestyle intervention in *n*orth India; *PREVENT-WIN* Study) was investigated at the outpatient department of National Diabetes, Obesity and Cholesterol Foundation (N-DOC), Diabetes Foundation (India) and Fortis-C-DOC Centre of Excellence for Diabetes, Metabolic Diseases and Endocrinology, New Delhi, India, from June 2015 to December 2018. The institutional ethics committee (Fortis-C-DOC Centre of Excellence for Diabetes, Metabolic Diseases and Endocrinology, New Delhi, India) approved the study. Individual informed consent was obtained. Clinical profile, anthropometry, body composition, biochemical investigations and carotid-femoral pulse wave velocity were performed in accordance with the relevant guidelines and regulation. Clinical Trial registration was done [clinical trial registration number; ClinicalTrials.Gov. NCT02513875 U.S. National Institutes of Health, USA].

Overweight/obese females aged 20–60 years who had prediabetes (as defined in definitions section) were included in the study. Exclusion criteria included those who received vitamin D and calcium supplementation in the previous six months, on any medication(s) within last one month which could potentially influence insulin secretion, insulin sensitivity, vitamin D and calcium metabolism, any severe acute or chronic illnesses, hyperparathyroidism, granulomatous disorders (e.g. sarcoidosis), patients who were on diuretics or calcium channel blockers and pregnant and lactating women.

### Randomization and intervention

**Figure 1:**
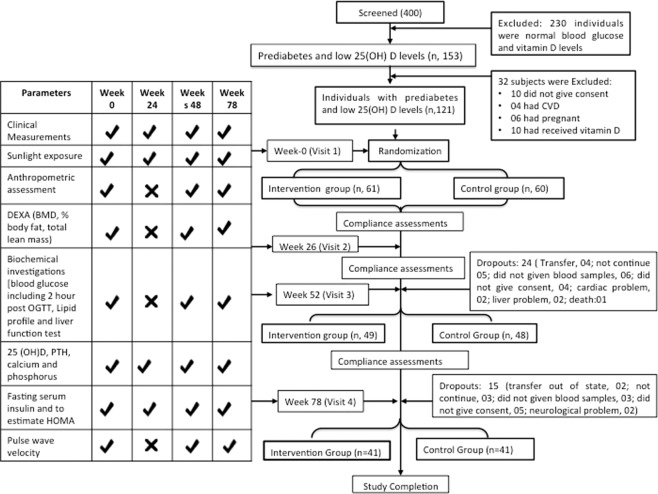
Consort Flow Diagram. PTH, Parathyroid hormone; HOMA-IR, Homeostasis Model Assessment Insulin resistance. *HbA1C estimation was done at week 0 and week 78.

This open-label randomized placebo-controlled trial was on 121 subjects with prediabetes and vitamin D deficiency **(**Fig. 1**)**. These individuals were allocated into one of the two groups by computer generated randomization list using variable block size. Group A (n, 61) received Vitamin D supplementation and oral calcium carbonate; and group B (n, 60) received placebo along with along calcium carbonate. For both groups, appropriate diet, and physical exercise counseling were given according to standard guidelines for Asian Indians^18^. Individuals were advised 45 min of brisk walking/or similar aerobic activity according to guidelines for Asian Indians^19^.

Supplementations with vitamin D and oral calcium were done in the following manner.

- Doses of cholecalciferol (commercial name, Calcirol, Cipla, India) 60,000IU (sachets, dissolved in half glass milk (120 ml) to be taken per orally) was given once per week for eight weeks to intervention group and Lactose Granules as placebo^20–22^.
- After every 24 weeks blood 25(OH) D levels were assessed. If subjects were still vitamin D deficient then supplementation of cholecalciferol 60,000 IU per week for eight weeks was repeated. If 25(OH)D level was normal, then oral cholecalciferol supplementation in doses of 200 IU per day was given as a maintenance dose.
- Equal doses of calcium carbonate (1 gm per day) was given orally with water to both the groups.

Compliance for the intervention was checked by taking following measures: (a) Biweekly telephonic calls, (b) Bi-monthly home visit by health workers (c) Six-monthly face-to-face sessions conducted by investigators, and (d) Log book maintenance by enrolled individuals for duration of sun exposure and vitamin D and calcium intake. Each individual was asked about the usual daily sun and skin exposure and the usual body parts exposed to sunlight during the hours of sun exposure (questionnaire reproduced in Supplement 1).

### Clinical measurements

Demographic and clinical profiles, medical history (personal and family), socioeconomic characteristics, skin exposure to sun and overall duration of sunlight exposure were determined with the use of questionnaire, which was pre-tested. Skin exposure was recorded by % of body surface area (face/hands, face/hands and arms, and face/hands and legs) exposed to sunlight. The duration of sunlight exposure (minutes/day) was assessed in the following manner; <5 minutes, 5–15 minutes, 15–30 minutes and >30 minutes (questionnaire reproduced in Supplement 1). Blood pressure was measured by a standard mercury sphygmomanometer over the right arm in sitting position.

Height was measured using a stadiometer. Height and body weight measurements were used to calculate the body mass index (BMI). Circumferences (waist, mid arm and neck) and skinfold measurements were taken from the right side of the body and were repeated three times at the same position and conditions. Average of the three readings were taken for the analysis. Skinfolds thickness (biceps, triceps, subscapular and suprailiac) were measured by a single observed using Lange skinfold calipers (Beta Technology Inc., Santa Cruz, CA, USA)^19^. The intra-observer variation in skinfold measurements were less than 5%, which was acceptable. Body composition of the individuals were estimated by DEXA [QDR-2000; Hologic, Waltham, MA, USA] and analyzed using the software version 7.20 as previously described^23^. FBG was performed after 12 h overnight fast by commercially available kit (RANDOX lab ltd, United Kingdom). A standard two hours oral glucose tolerance test (OGTT) was performed. Subject was instructed to take 75 g of carbohydrate in the form of anhydrous glucose mixed in 200 ml water orally over 5 minutes. Blood was drawn at 0 hour and then 2 hours after oral glucose load. Glycosylated hemoglobin (HbA1c) levels were measured (High Performance Liquid Chromatography, Biorad Labs, CA, USA). Lipid profile [total cholesterol (TC), serum triglycerides (TG), high-density lipoprotein cholesterol (HDL-c), and low-density lipoprotein cholesterol (LDL-c), liver function test [serum glutamic-oxaloacetic transaminase (SGOT), serum glutamate pyruvate transaminase (SGPT)], serum calcium and phosphorous were measured.

Fasting serum insulin was measured using a chemiluminescence immunoassay. Thereafter insulin resistance was recorded by two surrogate measures: fasting hyperinsulinemia and Homeostatic Model Assessment of Insulin Resistance (HOMA-IR). The value of HOMA-IR was calculated by the following equation: fasting insulin (µU/mL) X fasting blood glucose (mmol/L)/22.5. Serum 25(OH) D levels were measured by chemiluminescence method (DiaSorin 25-OH D assay, Stillwater, Minnesota, USA) as previously^5^. For this assay, the intra-assay coefficient of variation was 1.72% and the inter-assay coefficient was 2.01%. Fasting serum calcium levels were measured by colorimetric method (Randox Laboratory Ltd, Crumlin, UK). The intra-assay coefficient of variation was 3.14% and the inter-assay coefficient was 2.56%. Serum parathyroid hormone (PTH) was assayed by electro chemiluminescence method (Elecsys 2010, Roche Diagnostics, Mannheim, Germany) as previously^5^. The intra-assay coefficient of variation was 2.16% and the inter-assay coefficient was 2.41%. Arterial stiffness indices were assessed non-invasively by measuring carotid–femoral pulse wave velocity with a SphygmoCor apparatus (Atcor Medica, Australia).

### Definitions

Overweight and obesity were defined as BMI 23−24.9 kg/m^2^ and >25 kg/m^2^, respectively^24^. Blood pressure >130/85 mmHg (or on anti-hypertensive therapy) was defined as abnormal. Prediabetes was defined as FBG ≥100 mg/dl and <125.9 mg/dl, or 2-h post OGTT (after ingestion of 75 g anhydrous oral glucose mixed in 200 ml water) blood glucose levels ≥140 mg/dl and <200 mg/dl^25^. Serum 25(OH) D levels were categorized as follows; deficiency, <49·9 nmol/L, insufficiency, 50–75 nmol/L, and normal, >75 nmol/L^20,26^.

### Statistical analysis

Data were entered in an Excel spreadsheet (Microsoft Corp, Washington, USA). The distribution of demographic, clinical, medical history (personal and family), socioeconomic and behavioral characteristics, sun and skin exposure, anthropometry assessment, body composition, biochemical parameters and carotid-femoral pulse wave velocity variables were confirmed for approximate normality. Quantitative parameters were summarized by mean ± standard deviation, while qualitative variables were summarized as frequency (%). At baseline, the intervention and placebo groups were compared by Students’t’ test for continuous variables and Chi-square test for categorical variables. The compliance rate was calculated as the ratio between capsules used and capsules supplied for that time period.

Sample size was calculated according to previous study in South India^27^ has shown the progression of impaired glucose tolerance to T2DM is 40.5% without intervention. We expected that with vitamin D supplementation, it could be reduced up to 20%. To determine the change in outcome with an alpha error of 5%, 90% power and 20% dropouts, the effective sample size was 150 individuals (75 in intervention group and 75 in placebo group) in two groups.

The effects of vitamin D supplementation and placebo on the outcome variables were performed by both per protocol (PP) and as intention to treat (ITT). The last-value-carried-forward paradigm was used to fill in missing values to perform the ITT (61 intervention and 60 placebo group) analysis. The per protocol analysis for 81 subjects (41 intervention and 41 placebo group) who underwent randomization and completed 18 months of placebo and vitamin D supplementation was done. Overall effect size (95% CI) was computed using a generalized estimating equation (GEE) method. All statistical analyses were performed using Stata 14.0 statistical software (Stata Corp, TX, USA). p value < 0.05 was considered as statistically significant.

### Ethics approval

Institutional ethics committee of Fortis C-DOC Centre of Excellence for Diabetes, Metabolic Diseases and Endocrinology, Chirag Enclave, Nehru Place, New Delhi.

### Patient consent

Obtained.

## Results

### Baseline demographic, socioeconomic, and clinical profiles

There were no significant differences in demographic, socioeconomic, and family medical history between intervention and placebo groups at baseline (p > 0.05) **(**Table 1**)**. In personal medical history of the individuals, hypertension was significantly increased in placebo group as compared to intervention group (p = 0.05).

**Table 1:** Baseline profile.

Variables	Intervention (n, 61)	Placebo (n, 60)	p values
**Religion**			
Hindu	57 (95.0)	55 (91.8)	0.71
Muslim	2 (3.3)	4 (6.6)	
Christian	2 (1.7)	1 (1.6)	
**Marital status**			
Unmarried	5 (8.3)	2 (3.3)	0.28
Married	54 (90.0)	58 (96.7)	
Divorced	1 (1.7)	0	
**Qualification**			
Never attended school	12 (20.0)	19 (31.7)	0.39
Elementary	17 (28.3)	14 (23.3)	
Matriculation	15 (25.0)	14 (23.3)	
Higher secondary	11(18.3)	6 (10.0)	
Graduate	4 (6.7)	7 (11.7)	
Post Graduate	2 (1.7)	0	
**Employment**			
Housewife	40 (66.7)	36 (60.0)	0.23
Business	17 (26.7)	15 (25.0)	
Other services	4 (6.6)	9 (15.0)	
**Monthly income (US Dollar)**			
<156.5	12(20.0)	16 (26.7)	0.32
156.50–313.00	14 (23.3)	16 (26.7)	
314.00–469.50	17 (26.7)	8 (13.3)	
469.51–626.0	11(18.3)	14 (23.3)	
626.1–782.50	5 (8.3)	6 (10.0)	
>782.50	2 (1.2)	0	
**Tobacco intake**	1 (1.7)	3 (4.9)	0.21
**Alcohol intake**	1 (1.7)	1 (1.6)	0.99
**Personal medical history**			
Hypertension	17 (28.8)	27 (44.3)	0.05
Heart disease	0	3 (9.7)	0.07
**Family medical history**			
Diabetes	19 (31.7)	20 (32.8)	0.89
Hypertension	16 (26.7)	13 (21.3)	0.49
Heart disease	9 (15.6)	6 (9.9)	0.38

Results are shown as number (%). Chi-square test was done.

### Vitamin D levels after 24-weeks of supplementation

After 24 weeks of supplementation with Vitamin D, blood levels of 25(OH)D were as follows: sufficient (>75 nmol/L), 55.74% (34/61); insufficient (50–75 nmol/L), 37.70% (23/61) and deficient (<49·9 nmol/L), 6.6% (4/61).

### Sunlight exposure

In the final visit, skin exposure (face and hands) (p = 0.04) and duration of sun exposure (5–15 minutes/day) (p = 0.05) were significantly improved in intervention as compared to placebo group **(**Table 2**)**.

**Table 2:** Skin exposure to sunlight.

Variables	Week 0			Week 52			Week 78		
	Intervention (n, 61)	Placebo (n, 60)	p-value	Intervention (n, 61)	Placebo (n, 60)	p-value	Intervention (n, 61)	Placebo (n, 60)	p-value
**Skin exposure (n, %)**									
Face and hands	33 (54)	30 (50.0)	0.51	29 (47.5)	33 (55)	0.2	40 (65.6)	32 (53.4)	0.04
Face, hand and arms	19 (32)	20 (33.3)		21 (34.4)	19 (31.6)		18 (29.5)	16 (26.6)	
Face, hands and legs	9 (14)	10 (16.7)		11 (18.1)	8 (13.4)		03 (5)	12 (20)	
**Duration of sun exposure (minutes/day)**									
<5	10 (16.4)	9 (15)	0.55	12 (19.7)	14 (23.3)	0.5	9 (14.7)	20 (33.3)	0.05
5–15	41 (67.2)	42 (70)		39 (63.9)	31 (51.7)		38 (62.3)	19 (31.7)	
15–30	6 (9.9)	8 (13.3)		4 (6.6)	14 (23.3)		7 (11.5)	15 (25)	
>30	4 (6.5)	1 (1.7)		6 (9.8)	1 (1.7)		7 (11.5)	6 (10)	
**Time of sun exposure**									
Morning	33 (54.2)	33 (55)	0.89	33 (54.2)	36 (60)	0.37	50 (82)	33 (55)	0.66
Afternoon	25 (40.9)	24 (40)		27 (44.1)	19 (31.7)		9 (14.8)	20 (33.3)	
Evening	3 (4.9)	3 (5)		1 (1.6)	5 (8.3)		2 (3.2)	7 (11.7)	
Sun screen use	12 (24)	12 (23.0)	0.9	12 (24)	13 (25)	0.9	1 (2.7)	3 (6.8)	0.39

All values are shown as number (%). Intention-to-treat analyses has been conducted. p value < 0.05.

is statistically significant.

### Progression of prediabetes to diabetes and reversal to normoglycemia

**Figure 2:**
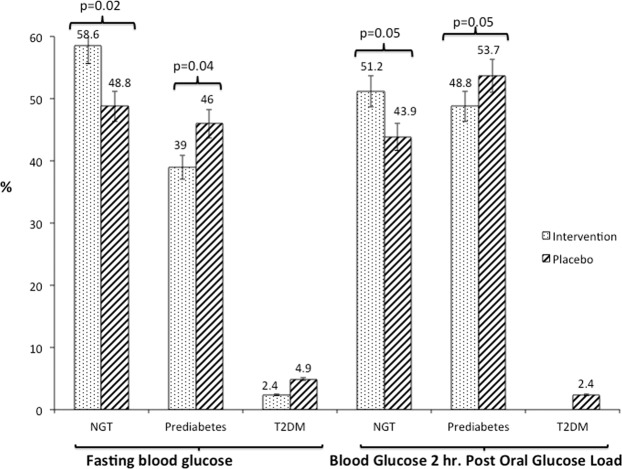
Progression of prediabetes to diabetes and reversal to normoglycemia after intervention. Vertical lines embedded in rectangles indicate standard error. P values are indicated on top of rectangles. NGT, normal glucose tolerance; T2DM, type 2 diabetes mellitus.

Changes in glycemic category based on FBG after intervention were as follows: intervention group: normal, 58.6%; impaired fasting glucose (IFG), 39%; and, T2DM, 2.4%; placebo group: normal fasting glucose, 48.8%; IFG, 46.3%; and T2DM, 4.9% **(**Fig. 2**)**. Changes in category of 2-hour glucose post OGTT after intervention were as follows; intervention group: normal glucose tolerance (NGT) 51.2% and prediabetes, 48.8%; placebo group: NGT, 43.9%; prediabetes, 53.7% and T2DM,2.4%.

### Intention-to-treat analysis of biochemical parameters

In ITT analysis, we found significant decrease in FBG [−5.0 (−12.6–2.4), p = 0.04], 2-hour blood glucose post OGTT [−11(−49.3–26.9), p = 0.02] and HbA1c [−0.41 (5.89, 6.55), p = 0.05) and increase 25(OH) D [7.5 (−6.0–20.9), p = 0.002] in intervention group as compared to placebo group at the end of the intervention **(**Tables 3–5**)**. We did not find any significant difference in PTH, calcium, phosphorus, fasting insulin and HOMA-IR between intervention and placebo groups (p > 0.05).Finally, we did not find any significant improvement between intervention group as compared to placebo group in any parameters at the end of the intervention as per protocol analysis (Table 3).

**Table 3:** Intention-to-treat (ITT) and per protocol (PP) analysis of biochemical parameters.

Variables		At 78 weeks		p value	Overall Effect Size (95 %CI)
		Intervention	Placebo		
FBG (mg/dl)	ITT	101.8 ± 11.9	106.9 ± 26.9	0.04	−5.0 (−12.6, 2.4)
	PP	107.4 ± 22.0	111.7 ± 30.7	0.46	−4.3 (−16.2, 7.5)
Haemoglobin A1c (%)	ITT	5.80 ± 1.05	6.21 ± 1.45	0.05	−0.41 (5.89, 6.51)
	PP	6.2 ± 1.21	6.18 ± 1.10	0.45	−0.03 (5.93, 6.46)
2-h blood glucose post OGTT (mg/dl)	ITT	138.4 ± 55.1	149.5 ± 61.9	0.02	−11.1 (−49.3, 26.9)
	PP	168.4 ± 81.6	162.6 ± 82.5	0.75	5.8 (−30.5, 42.1)
25(OH) D (nmol/l)	ITT	56.6 ± 12.6	49.1 ± 12.1	0.002	7.5 (−6.0, 20.9)
	PP	56.8 ± 32.1	48.7 ± 32.4	0.26	8.08 (−6.2, 22.3)
PTH (pg/ml)	ITT	37.9 ± 20.2	41.9 ± 23.8	0.05	−3.9 (−11.9, 3.9)
	PP	39.9 ± 18.2	40.8 ± 18.4	0.8	−0.89 (−8.9, 7.2)
Calcium (mg/dL)	ITT	9.3 ± 0.7	9.3 ± 0.7	0.98	−0.002 (−0.3, 0.3)
	PP	9.2 ± 0.3	9.2 ± 0.4	0.64	0.03 (−0.1, 0.2)
Phosphorus (mg/dL)	ITT	3.78 ± 0.5	3.89 ± 0.6	0.26	−0.10 (−0.3, 0.1)
	PP	3.8 ± 0.5	3.7 ± 0.4	0.5	0.069 (−0.2, 0.3)
Fasting insulin (μg/ml)	ITT	10.6 ± 4.8	10.1 ± 4.7	0.61	0.4 (−1.3, 2.2)
	PP	8.9 ± 4.0	9.1 ± 4.1	0.8	−0.14 (−1.9, 1.6)
HOMA-IR	ITT	1.20 ± 0.06	1.24 ± 0.04	0.09	−1.26 (−2.8, 0.3)
	PP	1.18 ± 0.04	1.19 ± 0.05	0.1	−1.18 (−2.4, 0.29)

ITT, Intention-to-treat (n, 61 for intervention group and n, 60 for placebo group); PP, per protocol (n, 41 for intervention group and n, 41 for placebo group); FBG, fasting blood glucose; OGTT, Oral glucose tolerance test; 25(OH) D, 25-hydroxy vitamin D; PTH, Parathyroid hormone; HOMA-IR, Homeostatic Model Assessment of Insulin Resistance. Values are expressed as mean  ± SD. p value < 0.05 is statistically significant.

**Table 4:** Biochemical profile.

Variables	Week 0			Week 26			Week 52			Week 78			Overall p value (Between the group	Overall Effect Size (95% CI) Using GEE Method
	Intervention (n, 61)	Placebo (n, 60)	p	Intervention (n, 61)	Placebo (n, 60)	p	Intervention (n, 61)	Placebo (n, 60)	p	Intervention (n, 61)	Placebo (n, 60)	p		
FBG (mg/dl)	111.9 ± 7.0	111.4 ± 7.6	0.2	110.9 ± 8.5	111.8 ± 7.9	0.5	108.4 ± 21.0	105.5 ± 17.5	0.04	101.8 ± 11.9	106.9 ± 26.9	0.04	0.87	0.38 (−4.4, 5.1)
2 hrs blood glucose post OGTT (mg/dl)	129.7 ± 38.3	124.5 ± 28.1	0.05	135.9 ± 35.6	126.5 ± 27.6	0.07	130.6 ± 38.3	124.5 ± 28.1	0.002	138.4 ± 55.1	149.5 ± 61.9	0.02	0.4	7.73 (−10.9, 26.4)
25(OH) D (nmol/l)	29.9 ± 5.9	32.1 ± 5.2	0.09	35.6 ± 6.5	32.5 ± 5.6	0.08	47.3 ± 11.2	42.2 ± 18.6	0.06	56.6 ± 12.6	49.1 ± 12.1	0.002	0.66	1.40 (−4.9, 7.7)
PTH (pg/ml)	58.5 ± 13.9	51.6 ± 11.0	0.05	45.6 ± 12.3	52.6 ± 10.3	0.001	37.0 ± 14.7	40.8 ± 19.5	0.07	37.9 ± 20.2	41.9 ± 23.8	0.05	0.45	2.59 (−4.2, 9.4)
Calcium (mg/dL)	9.0 ± 0.6	9.3 ± 0.5	0.3	9.1 ± 0.3	9.1 ± 0.7	0.6	9.5 ± 1.1	9.3 ± 0.4	0.9	9.2 ± 0.3	9.2 ± 0.34	0.64	0.43	0.14 (−0.21, 0.5)
Phosphorus (mg/dL)	3.6 ± 0.8	3.9 ± 0.7	0.8	3.2 ± 0.7	3.4 ± 0.5	0.4	3.6 ± 0.9	3.78 ± 0.8	0.4	3.8 ± 0.6	3.9 ± 0.6	0.26	0.39	−0.1 (−0.3, 0.1)
Fasting insulin (μg/ml)	14.2 ± 9.4	11.4 ± 7.5	0.07	12.3 ± 7.3	11.5 ± 8.7	0.08	10.7 ± 5.6	10.3 ± 4.8	0.7	10.6 ± 4.8	10.1 ± 4.7	0.61	0.09	−1.27 (−2.7, 0.2)
HOMA-IR	1.39 ± 0.08	1.81 ± 0.09	0.08	1.39 ± 0.08	1.81 ± 0.09	0.07	1.42 ± 0.09	1.38 ± 0.07	0.1	1.20 ± 0.06	1.24 ± 0.04	0.09	0.09	−1.26 (−2.8, 0.3)

ITT, Intention-to-treat. FBS, fasting blood glucose; OGTT, Oral glucose tolerance test; 25(OH) D, 25-hydroxy vitamin D; PTH, Parathyroid hormone; HOMA-IR, Homeostatic Model Assessment of Insulin Resistance; GEE, generalized estimating equation. p value < 0.05 is statistically significant.

**Figure 3:**
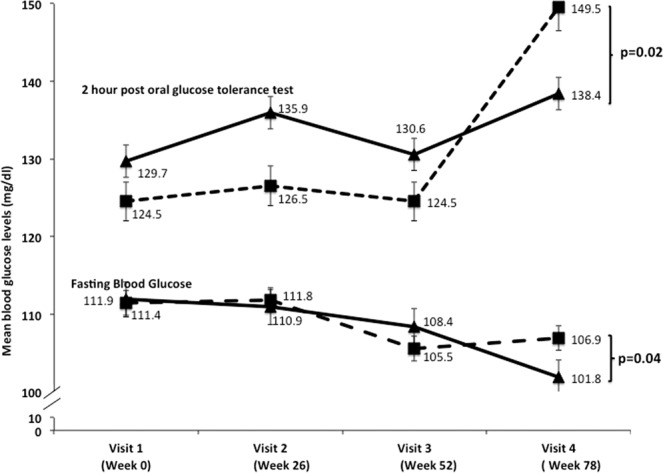
Comparison of blood glucose levels between intervention and control groups. Black triangles with black solid lines indicate intervention group and black squares with dotted line indicate placebo group. Actual value of each variable is given on the top of black triangle/black square. Vertical lines indicate standard error at each value. Top panel indicates values of blood glucose 2 hours after 75 gm oral anhydrous glucose while bottom panel indicates fasting blood glucose after 12 hours of overnight fast.

**Figure 4:**
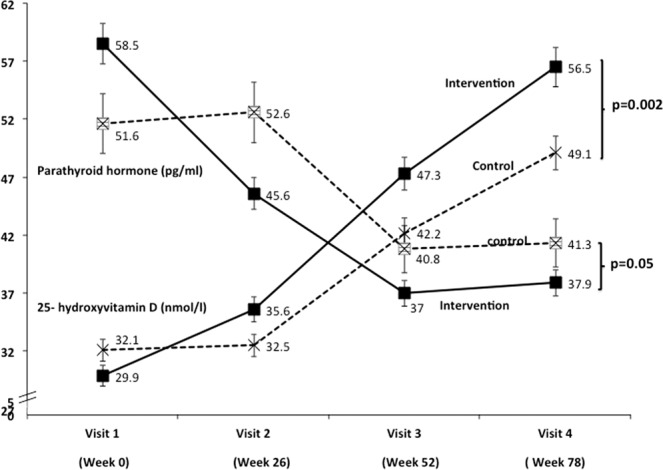
Comparison of 25 hydroxy vitamin D and parathyroid hormone levels between intervention and control groups. Black squares with black solid lines indicate intervention group and open black stars with dotted line indicate placebo group. Black squares with black solid lines in upper space indicate intervention group and open black stars with dotted line indicate placebo group. Actual value of each variable is given on the top black squares/open black stars. Vertical lines indicate standard error at each value.

Visit wise comparisons (ITT analysis) between the intervention and placebo groups has been shown in Table 4, Figs. 3 and 4. There were no significant differences in the baseline measurements of FBG, 2 h blood glucose post OGTT, HbA1c, fasting insulin, HOMA-IR, 25(OH) D, PTH, calcium, phosphorus, between intervention and placebo groups. PTH (p = 0.001) levels were significantly higher in placebo group at the end of six month. FBG (p = 0.04), 2-hour blood glucose post OGTT (p = 0.002) and PTH levels (p = 0.05) were significantly decreased and the levels of 25(OH)D were significantly increased (p = 0.002) in intervention group at the end of intervention. Further, no significant effect was seen on lipids and hepatic transaminases (serum TG, TC, HDL, LDL, VLDL, SGOT and SGPT) between intervention and placebo groups (Table 5).

**Table 5:** Lipids and hepatic transaminases.

Variables	Week 0		Week 52		Week 78		Overall p value (Between the groups)	Overall Effect Size (95% CI) Using GEE Method
	Intervention (n, 61)	Placebo (n, 60)	Intervention (n, 61)	Placebo (n, 60)	Intervention (n, 61)	Placebo (n, 60)		
Serum TG (mg/dl)	144.1 ± 75.1	156.1 ± 60.6	135.5 ± 51.9	154.9 ± 54.0	135.5 ± 51.9	154.9 ± 54.0	0.67	4.3 (−16.4, 24.7)
TC (mg/dl)	182.1 ± 42.9	188.8 ± 43.5	180.9 ± 35.4	194.0 ± 39.0	175 ± 35.1	198.7 ± 36.4	0.49	−5.3 (−20.6, 9.9)
HDL (mg/dl)	41.9 ± 11.4	42.5 ± 11.8	44.8 ± 9.7	43.7 ± 10.8	44.8 ± 9.7	43.7 ± 10.8	0.92	−0.17 (−3.6, 3.3)
LDL (mg/dl)	112.3 ± 34.4	120.9 ± 30.9	116.8 ± 26.7	123.5 ± 27.4	116.8 ± 26.7	123.5 ± 27.4	0.49	−3.9 (−15.3, 7.4)
SGOT (IU/L)	26.4 ± 9.5	34.4 ± 26.3	23.1 ± 11.1	29.8 ± 25.0	23.1 ± 11.1	29.8 ± 25.0	0.14	−4.4 (−10.2, 1.5)
SGPT (IU/L)	16.5 ± 8.9	22.0 ± 17.8	17.0 ± 6.8	18.1 ± 9.1	17.0 ± 6.8	18.1 ± 9.1	0.38	−1.7 (−5.5, 2.1)

Results are shown as mean ±  SD. p value < 0.05 is statistically significant. Effectiveness means that intention-to-treat analyses has been conducted. ^‡^TG, triglycerides; TC, total cholesterol; HDL, high-density lipoprotein; LDL, low-density lipoprotein, SGOT, serum glutamic pyruvic transaminase; SGPT; serum glutamic pyruvic transaminase; GEE, generalized estimating equation.

### Anthropometry, body composition and carotid-femoral pulse wave velocity profile

**Figure 5:**
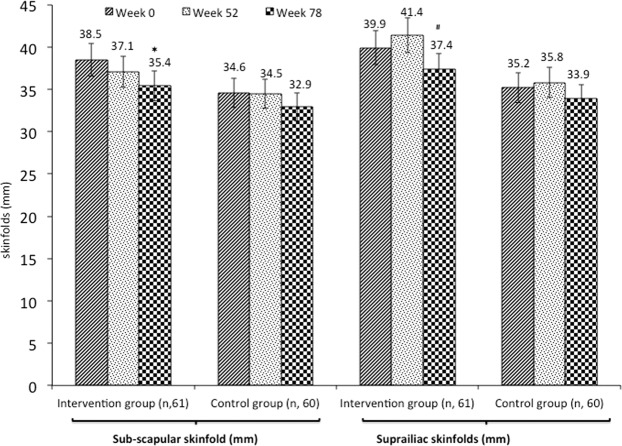
Comparison of sequential skinfolds measurements. Vertical lines embedded in rectangles indicate standard error. Actual values of each variable are given above the rectangles. *Significant reduction in visit III^rd^ as compared to visit I^st^. Significant reduction in visit III^rd^ as compared to Visit II^nd^.

Anthropometry (Table 6), body composition [total bone mineral density, % body fat, total fat (kg), total lean mass (kg) and % muscle mass (Table 7)] and carotid-femoral pulse wave velocity (Table 8) did not show any significant differences between intervention and placebo groups at the end of intervention. Subscapular skinfold (visit I^st^ compared to visit III^rd^) and suprailiac skinfold (visit II^nd^ compared to visit III^rd^) were significantly lower in intervention group (Fig. 5). After categorizing subjects into those having hypertension vs. non-hypertensive individuals, we did not find any difference in various parameters of carotid-femoral pulse wave velocity as measured by applanation tonometry [pulse wave velocity (m/s), aortic systolic pressure (mm Hg), aortic pulse pressure (mm Hg) augmentation index @75% HR, ejection duration and subendocardial viability ratio] in intervention and placebo groups (Supplement 2: Table 1).

**Table 6:** Anthropometric profile.

Variables	Week 0		Week 52		Week 78		Overall p value (Between the groups)	Overall Effect Size (95 %CI) Using GEE Method
	Intervention (n, 61)	Placebo (n, 60)	Intervention (n, 61)	Placebo (n, 60)	Intervention (n, 61)	Placebo (n, 60)		
Weight (kg)	70.3 ± 17.5	65.0 ± 11.7	70.3 ± 17.3	65.6 ± 10.5	71.1 ± 14.4	66.1 ± 11.0	0.39	2.92 (−3.8–9.7)
BMI (kg/m^2^)	31.1 ± 6.2	28.8 ± 3.9	30.5 ± 6.4	28.7 ± 3.0	30.6 ± 7.2	28.7 ± 3.7	0.26	−1.27 (−3.5–0.1)
**Circumferences (cm)**								
WC	93.9 ± 12.9	92.0 ± 8.9	91.3 ± 12.9	91.1 ± 7.9	90.2 ± 13.9	90.01 ± 8.2	0.22	2.34 (−1.4–6.1)
HC	100.7 ± 12.1	99.3 ± 8.8	99.3 ± 10.7	99.5 ± 8.3	98.6 ± 8.2	100.7 ± 10.5	0.83	0.4 (−3.3–4.1)
Mid -arm	26.9 ± 3.8	26.2 ± 4.2	26.9 ± 3.5	25.8 ± 3.7	26.4 ± 3.4	24.39 ± 1.1	0.47	−0.55 (−0.1–2.1)
Neck	31.7 ± 2.9	31.4 ± 3.1	30.7 ± 3.4	30.5 ± 3.3	30.1 ± 3.1	29.2 ± 2.3	0.20	0.73 (−0.4, 2)
**Skinfolds (mm)**								
Biceps	16.0 ± 8.2	14.0 ± 4.6	17.7 ± 7.8	15.7 ± 6.2	16.2 ± 7.1	15.1 ± 4.8	0.18	1.49 (−0.7,3.7)
Triceps	26.1 ± 8.1	24.4 ± 6.4	27.4 ± 8.1	29.04 ± 6.2	25.4 ± 7.1	24.3 ± 4.8	0.48	0.98 (−1.7, 3.7)

Results are shown as mean ±  SD. Effectiveness means that intention-to-treat analyses has been done. Values of subscapular and suprailiac skinfolds are given in Fig. 5. p value < 0.05 is statistically significant. BMI, body mass index; WC, waist circumference, HC, hip circumference; GEE, generalized estimating equation.

**Table 7:** Body composition by dual-energy x-ray absorptiometry.

Variables	Week 0		Week 52		Week 78		Overall p value (Between the groups)	Overall Effect Size (95 %CI) Using GEE Method
	Intervention (n, 61)	Placebo (n, 60)	Intervention (n, 61)	Placebo (n, 60)	Intervention (n, 61)	Placebo (n, 60)		
T score	0.43 ± 1.0	1.8 ± 1.3	0.3 ± 1.0	1.8 ± 1.1	0.2 ± 1.2	1.8 ± 1.1	0.15	0.1 (−0.4, 0.6)
Z Score	0.2 ± 0.8	0.1 ± 0.8	0.1 ± 0.8	0.1 ± 0.9	0.1 ± 0.8	0.1 ± 0.9	0.08	−0.3 (−0.7, 0.04)
Head BMD (g/cm^2^)	2.36 ± 0.03	2.3 ± 0.2	2.3 ± 0.3	2.3 ± 0.3	2.3 ± 0.3	2.3 ± 0.3	0.31	127.7 (−121.2, 376.8)
Arm BMD (g/cm^2^)	0.80 ± 0.1	0.84 ± 0.1	0.82 ± 0.1	0.83 ± 0.1	0.82 ± 0.	0.83 ± 0.1	0.21	−0.02 (−0.05, 0.01)
Leg BMD (g/cm^2^)	1.2 ± 0.1	1.2 ± 0.1	1.2 ± 0.1	1.2 ± 0.1	1.2 ± 0.1	1.2 ± 0.12	0.12	0.87 (0.8, 0.9)
Trunk BMD (g/cm^2^)	0.86 ± 0.1	0.91 ± 0.1	0.87 ± 0.1	0.90 ± 0.1	0.87 ± 0.1	0.90 ± 0.1	0.32	−0.02 (−0.05, 0.1)
Ribs BMD (g/cm^2^)	0.65 ± 0.1	0.68 ± 0.1	0.65 ± 0.1	0.67 ± 0.1	0.65 ± 0.1	0.67 ± 0.1	0.22	−0.01 (−0.03, 0.1)
Pelvis BMD (g/cm^2^)	1.0 ± 0.1	1.1 ± 0.1	1.0 ± 0.1	1.1 ± 0.1	1.0 ± 0.1	1.1 ± 0.1	0.6	1.4 (−0.7, 3.6)
Spine BMD (g/cm^2^)	1.0 ± 0.1	1.1 ± 0.1	1.0 ± 0.1	1.2 ± 0.1	1.0 ± 0.1	1.2 ± 0.1	0.24	0.9 (−1.7, 2.7)
Total BMD (g/cm^2^)	1.1 ± 0.1	1.1 ± 0.1	1.1 ± 0.1	1.1 ± 0.1	1.1 ± 0.1	1.1 ± 0.1	0.71	2.3 (−0.9, 5.2)
Total body fat (%)	45.9 ± 4.3	47.3 ± 5.8	46.1 ± 4.6	47.5 ± 5.9	46.1 ± 4.6	47.5 ± 5.9	0.87	0.6 (0.6, 1.4)
Total body fat (kg)	32.5 ± 5.5	32.04 ± 1.1	29.7 ± 5.8	33.4 ± 1.01	29.7 ± 5.8	33.4 ± 1.1	0.56	−1.8 (−6.5, 2.9)
Total lean mass (%)	65.3 ± 8.7	71.7 ± 16.5	66.1 ± 9.2	71.4 ± 15.9	66.1 ± 9.2	65.3 ± 8.7	0.45	1.6 (−0.7, 2.6)
Total lean mass (kg)	33.9 ± 3.9	34.5 ± 7.8	34.15 ± 4.3	35.6 ± 5.4	34.1 ± 4.3	35.6 ± 5.4	0.62	−0.9 (−1.7, 2.7)
BMC (Kg)	21.5 ± 3.8	21.9 ± 5.4	21.4 ± 3.8	22.6 ± 4.1	21.7 ± 3.9	22.6 ± 4.1	0.41	−58 (−199, 83.2)

Results are shown as mean ±  SD. p value < 0.05 statistically significant. Intention-to-treat analysis has been conducted. BMD, bone mineral density; BMC, bone mineral content; GEE, generalized estimating equation.

**Table 8:** Carotid-femoral pulse wave velocity.

Variables	Week 0		Week 52		Week 78		Overall p value (Between the groups)	Overall Effect Size (95 %CI) Using GEE Method
	Intervention (n, 61)	Placebo (n, 60)	Intervention (n, 61)	Placebo (n, 60)	Intervention (n, 61)	Placebo (n, 60)		
Pulse wave velocity (m/s)	7.1 ± 1.6	7.4 ± 2.2	6.8 ± 1.9	7.4 ± 2.2	6.8 ± 1.9	7.4 ± 2.2	0.55	−1.6 (−4.5, 9.1)
ASP (mmHg)	112.1 ± 5.1	112.3 ± 5.4	112.1 ± 2.8	111.8 ± 5.3	112.01 ± 2.8	111.8 ± 5.38	0.52	1.45 (−0.7,3.6)
APP (mmHg)	31.5 ± 3.4	30.6 ± 4.33	31.77 ± 3.4	30.6 ± 4.3	31.7 ± 3.4	30.6 ± 4.33	0.56	−1.9 (−6.5, 2.4)
Alx@HR75 (%)	29.0 ± 8.06	26.5 ± 8.99	29.5 ± 7.9	27.1 ± 8.3	29.5 ± 7.9	27.1 ± 8.3	0.34	−1.6 (−6.5, 2.6)
Ejection duration (%)	39.1 ± 3.6	39.4 ± 4.23	41.2 ± 12.5	39.5 ± 4.1	41.2 ± 5.3	41.4 ± 6.5	0.75	−0.9 (−1.5, 2.9)
SEVR (%)	136.4 ± 21.2	136 ± 23.2	135 ± 24.4	135.7 ± 22.8	135.1 ± 24.4	135.7 ± 22.8	0.96	−3.9 (−15.5, 7.5)

Results are shown as mean ±  SD. p value < 0.05 statistically significant. Intention-to-treat analysis has been conducted. ASP, aortic systolic pressure (mm Hg); APP, aortic pulse pressure (mm Hg) Alx@75% HR, augmentation index @75% HR, SEVR, sub-endocardial viability ratio; GEE, generalized estimating equation. Further data provided in Supplementary Table 1.

## Discussion

In this carefully designed randomized control trial of 18 months duration, we show significant decrease in the following; FBG, 2-h blood glucose post OGTT, HbA1c, and truncal subcutaneous fat (as measured by skinfolds) with vitamin D intervention. Further, significantly greater number of subjects reverted to normoglycemia in intervention group. These findings have been reported exclusively in vitamin D deficient women with prediabetes with longest trial duration (compared to all studies done till date) for the first time. Strength of the study is that it is carefully executed randomized controlled trial.

The effect of vitamin D supplementation on insulin resistance continues to be researched and debated. There is growing evidence that vitamin D deficiency may be a contributing factor in the pathogenesis of type 1 and type 2 diabetes. Vitamin D regulates the function of calbindin, a systolic calcium-binding protein found in pancreatic β-cells, and acts as a modulator of depolarization-stimulated insulin secretion via regulation of intracellular calcium^28^). Additionally, administration of vitamin D restores glucose-stimulated insulin secretion and promotes β-cell survival by modulating the generation and effects of cytokines^29^. Other mechanisms associated with vitamin D and diabetes include enhancement of insulin responsiveness for glucose transport, and improvement of systemic inflammation^30^.

However, in human studies, effects of vitamin D supplementation on insulin sensitivity are not consistent; beneficial in healthy Japanese (12 month study)^31^ and centrally obese Asian Indian (8 weeks study)^32^, but not seen in elderly overweight Lebanese (12 month study)^33^ populations or in our current study (HOMA_IR)on women with prediabetes with longer duration of follow up. Further, there were no effects of vitamin D supplementation on markers of inflammation or endothelial function in healthy South Asian women (4 weeks study) living in UK^34^. In a trial on insulin resistant migrant South Asian women living in New Zealand, improvement in vitamin D status resulted in improved insulin resistance and sensitivity, but no change in insulin secretion was recorded^35^.

Similarly, the results of studies on effects of Vitamin D replacement on glycemia have been inconsistent. Results from Women’s Health Initiative Study did not show the positive effect of daily supplementation of 1000 mg of calcium and 400 IU of Vitamin D for a period of 7 years in preventing the risk of T2DM^36^. A systematic review and meta-analysis of twenty-three randomized controlled trials (vitamin D intervention *vs*. placebo; n, 1797) showed no significant effect in change of HbA_1c_ with Vitamin D in patients with T2DM. However, a significant effect of vitamin D supplementation was seen on FBG in 4 studies with a mean baseline HbA_1c_ ≥ 8%^37^. Effects of vitamin D supplementation on glycemia in different ethnic group with prediabetes have been inconsistent too; no improvements seen in in African Americans^38^, Canadians^39^ and Norwegians^40^, and improvement shown in Asian Indians^22^.

Literature review and meta-analysis of all existing studies using vitamin D supplementation have been published recently; these show only 6 studies where vitamin D has been used in individuals with prediabetes and low vitamin D. Only three (two from USA, one from India) of these were of 12 months duration. Interestingly summary findings from this meta-analysis indicate that benefits from vitamin D supplementation on insulin resistance occurs when administered for longer duration (more than 6 months) and with calcium, both conditions fulfilled in our study. Interestingly, although our study had longest supplementation duration, no effect on fasting insulin and HOMA-IR were seen.

Intervention with vitamin D supplementation Asian Indians with prediabetes are fewer, but benefits have been more consistent. In an open label randomized prospective study (12 month follow up), Dutta *et al*.^22^, from western India, reported that vitamin D supplementation in subjects with prediabetes lead to lower progression to diabetes and higher reversal to normoglycemia. Interestingly, in this study increase in blood vitamin levels was not substantial *vs* our data which show major increase in vitamin D levels in intervention as well as control groups. In an another one-year open labelled randomized study on ethnically homogeneous Kashmiri population, all measures of glycemia improved with vitamin D supplementation group^22^. Our study, conducted over longer period (18 months) shows improvement of glycemia with some differences. Improvements in fasting blood glucose and 2-hour blood glucose (OGTT) occurred only in last 6 months of follow up, which may mean effects of vitamin D on glycemia may occur over long periods, and that in our patients no significant effects on serum insulin or HOMA-IR were seen. Interestingly, levels of vitamin D rose significantly more in last 26 weeks of the study, time period when significant effect on glycemia was also seen. It is important to note that beneficial effects of vitamin D supplementation on insulin sensitivity was seen even after 8 weeks intervention in healthy subjects residing is same geographical region (New Delhi) previously^32^, and over one year in individuals with prediabetes in western India^22^. An additional research point is that, in difference to previous studies on Asian Indians, we included detailed analysis of body composition including skinfold thickness, lean mass and bone mineral density using multiple measures of body composition, and also studied carotid-femoral pulse wave velocity (non-invasive marker of atherosclerosis).

These inconsistent data of vitamin D supplementation on measures of glycemia and insulin sensitivity in previous studies may be due to several factors; use of varying doses of vitamin D and different duration of studies, different age range of subjects, and possible effects of ethnicity. If we analyse studies done on Asian Indians, more consistent results on glycemia, but divergent results on insulin sensitivity were seen. It is hard to interpret these observations without further studies, but it is possible that vitamin D supplementation may work better on glycemic parameters in Asian Indians, who are majorly vitamin D deficient (more than other races according to some studies) and in whom, prediabetes rapidly converts to diabetes.

Vitamin D deficiency has been associated with obesity, and this association is believed to be complex. In a systematic review and meta-analysis, no effect of vitamin D or calcium supplementation on BMI or fat mass was seen^41,42^. Alternatively, there is paucity of data of effects of vitamin D supplementation on body composition in detail. In a double-blind study, 52 obese subjects (aged 18 to 50 years) with plasma 25(OH) D <50 nmol/l were randomized to 26 weeks of treatment with 7000IU vitamin D daily or placebo. In this short-term study, body composition evaluation was done with DEXA, MRI and MR spectroscopy. Intervention with vitamin D did not result in changes in body fat, subcutaneous abdominal fat, visceral adipose tissue (VAT), intra-hepatic or intramyocellular triglycerides as compared with placebo^43^. In a study on sixty Iranian patients with T2DM given vitamin D supplementation for 12 weeks, decrease in waist circumference, body fat, truncal fat, and intra-abdominal VAT was seen, more so in carriers of the AA genotype of VDR-Cdx-2^44^. In part, our results of lack of effects of vitamin D on total body fat are akin to some previous data, while reduction of subcutaneous fat (decrease in skinfold thickness) is a novel finding for Asian Indians. Interestingly, abdominal subcutaneous adipose tissue is thicker and appears to be another major determinant (apart from intra-abdominal VAT) of insulin resistance in South Asians/Asian Indians^45^. While we did not measure intra-abdominal VAT, comparative data between Europeans and South Asians in Canada showed that VAT remained negatively (P < 0.05) associated with plasma 25(OH) D concentrations modulated by ethnicity^1^.In a systematic review no significant effect of vitamin D supplementation on muscle mass or muscle power was reported^46^, and our data are consistent with those observations. Similarly, we show no benefit of vitamin D supplementation on a bone mineral density, although a previous meta-analysis showed a small benefit of vitamin D supplementation on BMD at the femoral neck (with heterogeneity among trials)^47^. Vitamin D administration in the subjects in the current study did not produce any toxicity. Specifically, neither symptoms of hypercalcemia were seen, nor biochemical evidence of hypercalcemia was noted in serial measurement of serum calcium.The current study had a few limitations. We could not recruit required number of subjects as per power calculations because of financial constraints. Further, larger longitudinal studies are warranted to study interplay between vitamin D deficiency, obesity, insulin resistance, and T2DM in Asian Indians.

## Conclusion

In this study, we show significant decrease in fasting blood glucose, 2-h blood glucose (post OGTT), HbA1c, and truncal subcutaneous fat with vitamin D supplementation over 78 weeks in overweight/obese prediabetic and vitamin D deficient Asian Indian women.

## Supplementary information

Supplementary Information

Supplementary Information 2

## Data Availability

All data generated or analysed during this study are included in this article.
