# Author Correction: Vitamin D Supplementation in Overweight/obese Asian Indian Women with Prediabetes Reduces Glycemic Measures and Truncal Subcutaneous Fat: A 78 Weeks Randomized Placebo-Controlled Trial (*PREVENT-WIN* Trial)

**DOI:** 10.1038/s41598-020-67064-9

**Published:** 2020-06-12

**Authors:** Surya Prakash Bhatt, Anoop Misra, Ravindra Mohan Pandey, Ashish Datt Upadhyay, Seema Gulati, Namrata Singh

**Affiliations:** 1 Diabetes Foundation (India), Safdarjung Development Area, New Delhi, 110016 India; 2 National Diabetes Obesity and Cholesterol Foundation (N-DOC), Safdarjung Development Area, New Delhi, 110016 India; 3 Department of Pulmonary Medicine and Sleep Disorders, All India Institute of Medical Sciences, Ansari Nagar, New Delhi 110029 India; 4 Biostatistics, All India Institute of Medical Sciences, Ansari Nagar, New Delhi 110029 India; 5 Fortis C-DOC Center of Excellence for Diabetes, Metabolic Diseases, and Endocrinology, B-16, Chirag Enclave, New Delhi India

Correction to: *Scientific Reports* 10.1038/s41598-019-56904-y, published online 14 January 2020

This Article contains an error in the Methods section under the subheading ‘Randomization and intervention’ where,

“(a) Doses of cholecalciferol (commercial name, Calcirol, Cipla, India) 60,000IU (sachets, dissolved in half glass milk (120 ml) to be taken per orally) was given once per week for eight weeks to intervention group and Lactose Granules as placebo^20,21,22^.”

should read:

“(a) Doses of cholecalciferol (commercial name, Calcirol, Cadila, India) 60,000IU (sachets, dissolved in half glass milk (120 ml) to be taken per orally) was given once per week for eight weeks to intervention group and Lactose Granules as placebo^20,21,22^.”

