# Supplementary material for: Vitamin D Supplementation in Overweight/obese Asian Indian Women with Prediabetes Reduces Glycemic Measures and Truncal Subcutaneous Fat: A 78 Weeks Randomized Placebo-Controlled Trial (PREVENT-WIN Trial)
Source: Sci Rep. 2020 Jan 14;10:220. doi: 10.1038/s41598-019-56904-y (PMC6959323; doi:10.1038/s41598-019-56904-y)
Supplement: Supplementary file 1 — Supplementary Information [file 41598_2019_56904_MOESM1_ESM.doc]

Title: Vitamin D Supplementation in Overweight/obese Asian Indian Women with Prediabetes Reduces Glycemic Measures and Truncal Subcutaneous Fat: A 18-month Randomized Controlled Trial (*PREVENT-WIN* Trial).

Authors; Surya Prakash Bhatt^1,2,3,5^, Ph.D.; Anoop Misra^1,2,5^, MD; Ravindra Mohan Pandey^4^,Ph.D; Ashish Datt Upadhyay^,4^, Ph.D.; Seema Gulati^1,2^, PhD; Namrata Singh^1,2^, MSc

^1^Diabetes Foundation (India), Safdarjung Development Area, New Delhi 110016, India.

^2^National Diabetes Obesity and Cholesterol Foundation (N-DOC), Safdarjung Development Area, New Delhi 110016, India.

^3^Department of Pulmonary Medicine and Sleep Disorders, and ^4^Biostatistics, All India Institute of Medical Sciences, Ansari Nagar, New Delhi 110029, India

^5^Fortis C-DOC Center of Excellence for Diabetes, Metabolic Diseases, and Endocrinology, B-16, Chirag Enclave, New Delhi, India

Address for correspondence and reprint request to:

Professor Anoop Misra

Chairman, Fortis-CDOC Center of Excellence for Diabetes, Metabolic Diseases and Endocrinology, B-16, Chirag Enclave, New Delhi, India.

Ph: 91-11-4277-6222 (Ext: 5030); Fax: 91-11-4277-6221; Email: [anoopmisra@gmail.com](mailto:anoopmisra@gmail.com)

**ANNEXURE**

**PROFORMA**

**PREVENTION OF TYPE 2 T2DM IN WOMEN WITH PREDIABETES WITH VITAMIN D SUPPLEMENTATION AND LIFESTYLE INTERVENTION IN NORTH INDIA (PREVENT-WIN STUDY)**

**Name of the site-**

**Interviewer Name-**

**Date-**

**Section -1 (Background Information)**

1.1 ID No- (cases-1, controls-2)

1.2 Name- ___________________________

1.3 Age - Sex- (Male-1, Female-2)

1.4 Address-_____________________________________

_____________________________________

Phone no- __________________ Mobile no- ____________e-mail ID-__________

1.5 Religion- _______________________

1.6 Which is your state of origin?:___________________________________

1.7 What was your approximate birth weight? ________________

1.8 Are You:

Married -1

Divorced -2

Widowed -3

Separated -4

Never been married -5

A member of an unmarried couple -6

1.9 If female, did you have a baby weighing more then 9lbs (4kgs) at birth?(yes-1,No-2)

1.10 Educational qualification-

1-Never attended school

2-Grade 1 to 8 (elementary)

3- Grades 9 to 10(matriculation)

4-Higher secondary

5 - Some college or diploma

6- Graduate

7-Post graduate

8-Professional

Self- Spouse-

1.11 Present occupation- ________________________________________

1.12 Is your monthly household income from all sources :

Less than Rs 10000 -1

Rs 10001 to Rs 25000 -2

Rs 25001 to Rs 50000 3

Rs 50001 to Rs 1,00,000 -4

More then Rs 1,00,000 -5

**SECTION 2: Eligibility Criteria**

**2.1 For Cross sectional Study**

2.1.1 Subjects will be considered eligible to participate in the study if they meet the following inclusion criteria:

**Inclusion Criteria**

Yes

No

1. Age 20-60 years healthy adult females.

2.1.2 Subjects will be considered ineligible to participate in the study if they meet one or more of the following exclusion criteria:

**Exclusion Criteria:**

1. Received Vitamin D/calcium supplementation in the previous six months.

Yes

No

2. On any medication within last one month which could potentially influence insulin secretion, insulin sensitivity, vitamin D or calcium metabolism (eg. metformin, thiazolidione, steroids etc.) and on any medication that activate steroid and xenobiotic receptor and drugs used in transplantation (e.g. steriods, calcitonin etc.)

Yes

No

3. Pregnancy & lactation at time of study.

Yes

No

4. Severe end organ damage or chronic diseases: renal/hepatic failure, any malignancy, major systemic illness etc.

Yes

No

Yes

No

5. Known case of HIV infection.

6. Known case of diabetes mellitus and endocrinal disorders.

Yes

No

**2.2 For Prospective Study**

2.2.1 Subjects will be considered eligible to participate in the study if they meet the following inclusion criteria:

**Inclusion Criteria:**

Yes

No

1. Fasting blood glucose ≥100mg/dl and <126mg/dl **or**

2. 2-h plasma glucose ≥140mg/dl and <200mg/dl (after ingestion of 75 g glucose) **and**

Yes

No

3. Level of 25 hydroxy vitamin D <30ng/dl.

Yes

No

Exclusion Criteria:

1. Received Vitamin D/calcium supplementation in the previous six months.

Yes

No

2. On any medication within last one month which could potentially influence insulin secretion, insulin sensitivity, vitamin D or calcium metabolism (eg metformin, thiazolidione, steroids etc) and on any medication that activate steroid and xenobiotic receptor and drugs used in transplantation (e.g. steriods, calcitonin etc.)

Yes

No

3. Pregnancy & lactation at the time of study.

Yes

No

4. Severe end organ damage or chronic diseases: renal/ hepatic failure, any malignancy, nephrotic syndrome, malabsorption etc.

Yes

No

5. Known case of HIV infection.

Yes

No

6. Primary or tertiary hyperparathyroidism, granulomatous disorders (e.g. sarcoidosis) and any lymphomas

Yes

No

Yes

No

7. Known case of diabetes mellitus.

**Section 3: Health Background**

**Tobacco use**

3.1 Do you consume tobacco in any form?

Not at all -0

Sometimes -1

Regularly -2

Stopped (>6 months) -3

3.2 Quantity and duration of use

| Type of tobacco | Duration of use (in yrs) | Number of days use per week | Number of use per day | When Stopped (in years) |
| --- | --- | --- | --- | --- |
| Cigarettes |  |  |  |  |
| Beedi |  |  |  |  |
| Cigars |  |  |  |  |
| Hukka |  |  |  |  |
| Chewing tobacco |  |  |  |  |
| Pan with zardha |  |  |  |  |
| Gutka |  |  |  |  |
| Betel |  |  |  |  |
| Snuff |  |  |  |  |

**Alcohol use**

3.3 Do you consume alcohol in any form?

Not at all -0

Sometimes -1

Regularly -2

Stopped (>6 months) -3

3.4 Quantity and duration of use

| **Type of alcohol** | **Duration of use (in years)** | **Number of days of use** | | | **Numbers of drink per day** | **When stopped (in years)** |
| --- | --- | --- | --- | --- | --- | --- |
|  |  | **Per week** | **Per month** | **Per year** |  |  |
| Beer |  |  |  |  |  |  |
| Wine |  |  |  |  |  |  |
| Spirit |  |  |  |  |  |  |
| Whiskey |  |  |  |  |  |  |
| Toddy |  |  |  |  |  |  |
| Rum |  |  |  |  |  |  |
| Brandy |  |  |  |  |  |  |
| Gin |  |  |  |  |  |  |
| Vodka |  |  |  |  |  |  |
| Arrack |  |  |  |  |  |  |

*Note- 1 drink =30 ml of spirit, whiskey, toddy, rum, brandy, gin, vodka, arrack (or) 125 ml of wine (or) 325 ml of beer*.

3.5 Personal History**-**

| **Disease** | **Yes/No** | **Duration** | **Medication** |
| --- | --- | --- | --- |
| Hypertension |  |  |  |
| Heart Disease |  |  |  |
| Thyroid Dysfunction |  |  |  |
| Liver disease |  |  |  |
| Any other complication |  |  |  |

3.6 Family History**-**

| **Disease** | **Relation** | **Age When developed** |
| --- | --- | --- |
| Obesity |  |  |
| Diabetes |  |  |
| Hypertension |  |  |
| Heart Disease |  |  |
| Thyroid dysfunction |  |  |
| Liver disease |  |  |

**SECTION 4: SUN EXPOSURE**

- 1. Skin Exposure:

Face/hands

Face/hands and arms

Face/hands and legs

- 1. Duration of Sun Exposure (minutes/day):

< 5 min

5-15 min

15-30 min

If more then 30 min then duration ……….

4.3 Duration of outdoors during day light

In summers ………..hr/day

In winters ……...hr/day

4.4 Use of suns screen:

In summers …….. days/week

In winters …….. days/week

4.5 Measurement of Light at Premises (by Lux meter)

In summers…… lux

In Winter …… lux

**Section 5 (clinical examination & Biochemical Test)**

5.1 Acanthosis Nigricans -Yes/No

Buffalo Hump -Yes/No

Double Chin -Yes/No

5.2 Vital signs

Resting pulse rate ( / min): ____________

Blood pressure (mm/Hg): Sitting: ___________

(Two readings 10 min apart) Standing: _________

Chest: _________ CVS:__________ Abdomen:______________________

CNS:__________ Power:__________ DTR:______

**5.3. Biochemical Tests**

| **Investigation** | **Result** | **Units** |
| --- | --- | --- |
| **Glycaemic Profile** | | |
| Fasting Blood Glucose (0hr) |  | mg/100ml |
| Fasting Insulin (0hr) |  | uU/ml |
| Blood Glucose (2hr) |  | mg/100ml |
| Fasting Insulin (2hr) |  | uU/ml |
| **Lipid Profile** | | |
| Serum Cholesterol |  | mg/dl |
| HDL cholesterol |  | mg/100ml |
| Triglycerides |  | mg/100ml |
| VLDL |  | mg/dl |
| LDL |  | mg/dl |
| Parathyroid Harmone |  |  |
| Calcium |  |  |
| Phosphorus |  |  |
| 25 (OH)D |  | ng/ml |

**Section-6 (Physical measurement)**

Height (cm):____________ Weight (kg):__________ BMI (kg/m^2^):________

|  | 1 | 2 | 3 | Average |
| --- | --- | --- | --- | --- |
| Waist circumference (cm) |  |  |  |  |
| Hip circumference (cm) |  |  |  |  |
| Waist/Hip Ratio |  |  |  |  |
| Waist/Height Ratio |  |  |  |  |
| Mid arm circumference(cm) |  |  |  |  |
| Neck circumference(cm) |  |  |  |  |
| Biceps (mm) |  |  |  |  |
| Triceps (mm) |  |  |  |  |
| Sub scapular (mm) |  |  |  |  |
| Suprailliac (mm) |  |  |  |  |

**Body Composition Analyser**

Bioelectrical Impedance (Ω): ____ Fat mass (kg):____

Fat free mass (kg):______

Total body water (kg):__________ Fat percentage:________

**Section-7 Dietary Proforma (3-d dietary recall)**

7.1 Type of Family- (1=joint,2=nuclear)

Total No of people eating from the same kitchen- (adults:__ children:__)

7.2 Dietary habits- A) Vegetarian (no eggs)

B) Vegetarian (with eggs)

C) Non-vegetarian

7.3 How often do you eat outside your home or purchase food from outside. Type of facility, how often (times/week)

Sit in restaurant- ______________ Dhaba-_______________

Fast food - ______________ Other- _______________

**7.4 Fats and oil consumption-**

Total consumption of (kg) Monthly Daily

Ghee (pure)

Purchased ______ ______

Home made ______ ______

Refined oil

Oil 1 ( ______ ) ______ ______

Oil2 (______ ) ______ ______

Vanaspati ____ ______

Margarine ______ ______

Butter ______ ______

Cheese ______ ______

Processed cheese

Cottage cheese (paneer)

-Home made _____ ______

-Commercial ____ ______

7.5 **Food frequency questionnaire-**

| **Food Stuff** | **Portion size (at a time)** | **Frequency** | | | | **Amount**  **(g)** |
| --- | --- | --- | --- | --- | --- | --- |
|  |  | **Daily** | **Weekly** | **Monthly** | **Occasionally** |  |
| **NUTS AND OILSEEDS** |  |  |  |  |  |  |
| Badaam |  |  |  |  |  |  |
| Dates |  |  |  |  |  |  |
| Groundnut(……..) |  |  |  |  |  |  |
| Kajoo |  |  |  |  |  |  |
| Nariyal(…………) |  |  |  |  |  |  |
| Pista |  |  |  |  |  |  |
| Raisins |  |  |  |  |  |  |
| Walnuts |  |  |  |  |  |  |
| **FRUIT** |  |  |  |  |  |  |
| Apple |  |  |  |  |  |  |
| Banana |  |  |  |  |  |  |
| Custard apple |  |  |  |  |  |  |
| Grapes |  |  |  |  |  |  |
| Guava |  |  |  |  |  |  |
| Lime |  |  |  |  |  |  |
| Litchi |  |  |  |  |  |  |
| Mango |  |  |  |  |  |  |
| Melon, musk |  |  |  |  |  |  |
| Melon, water |  |  |  |  |  |  |
| Orange |  |  |  |  |  |  |
| Papaya |  |  |  |  |  |  |
| Peach |  |  |  |  |  |  |
| Pine apple |  |  |  |  |  |  |
| Plum |  |  |  |  |  |  |
| Pomegranate |  |  |  |  |  |  |
| Sapota |  |  |  |  |  |  |
| **NON VEG** |  |  |  |  |  |  |
| Beef(…………..) |  |  |  |  |  |  |
| Chicken(……….) |  |  |  |  |  |  |
| Egg(………..) |  |  |  |  |  |  |
| Fish(…………) |  |  |  |  |  |  |
| Mutton(………..) |  |  |  |  |  |  |
| Pork |  |  |  |  |  |  |
| **MILK AND ITS PRODUCTS** |  |  |  |  |  |  |
| Buttermilk |  |  |  |  |  |  |
| Cheese(……….) |  |  |  |  |  |  |
| Curd |  |  |  |  |  |  |
| Milk(………………) |  |  |  |  |  |  |
| Milk, double toned |  |  |  |  |  |  |
| Milk, skimmed |  |  |  |  |  |  |
| Milk, toned |  |  |  |  |  |  |
| Skimmed milk powder |  |  |  |  |  |  |
| Whole milk, powder |  |  |  |  |  |  |
| **BEVERAGES** |  |  |  |  |  |  |
| Juice(……….) |  |  |  |  |  |  |
| Shake(………) |  |  |  |  |  |  |
| Lassi(………) |  |  |  |  |  |  |
| Tea |  |  |  |  |  |  |
| Coffee |  |  |  |  |  |  |
| Cold drink(………) |  |  |  |  |  |  |
| **VEGETABLES** |  |  |  |  |  |  |
| Roots & Tubers |  |  |  |  |  |  |
| Green Leafy |  |  |  |  |  |  |
| Others |  |  |  |  |  |  |
| **Pulses** |  |  |  |  |  |  |
| With Husk |  |  |  |  |  |  |
| Without Husk |  |  |  |  |  |  |
| **MISSCELLANEOUS** |  |  |  |  |  |  |
| Burger |  |  |  |  |  |  |
| Chocolate |  |  |  |  |  |  |
| Kachori |  |  |  |  |  |  |
| Mathri |  |  |  |  |  |  |
| Corn flakes |  |  |  |  |  |  |
| Pizza |  |  |  |  |  |  |
| Samosa |  |  |  |  |  |  |
| Vada |  |  |  |  |  |  |
| Wafers |  |  |  |  |  |  |
| Ice-cream |  |  |  |  |  |  |
| Soup(…………) |  |  |  |  |  |  |
| Papad |  |  |  |  |  |  |
| Pickle |  |  |  |  |  |  |
| Popcorn |  |  |  |  |  |  |
| Savory/Namkeen |  |  |  |  |  |  |
| Pakoda |  |  |  |  |  |  |
| Sweet |  |  |  |  |  |  |
| Sugar |  |  |  |  |  |  |
| Candy |  |  |  |  |  |  |
| South Indian |  |  |  |  |  |  |
| Pastry |  |  |  |  |  |  |
| Cookies |  |  |  |  |  |  |
|  |  |  |  |  |  |  |

**7.6 Weekdays -1**

| **Meal time** | **Menu** | **Serving Size** | **Ingredients** | **Amount (gm)** |
| --- | --- | --- | --- | --- |
|  |  |  |  |  |
|  |  |  |  |  |
|  |  |  |  |  |
|  |  |  |  |  |
|  |  |  |  |  |

**Weekdays -2**

| **Meal time** | **Menu** | **Serving Size** | **Ingredients** | **Amount (gm)** |
| --- | --- | --- | --- | --- |
|  |  |  |  |  |
|  |  |  |  |  |
|  |  |  |  |  |
|  |  |  |  |  |
|  |  |  |  |  |

**Weekend**

| **Meal time** | **Menu** | **Serving Size** | **Ingredients** | **Amount (gm)** |
| --- | --- | --- | --- | --- |
|  |  |  |  |  |
|  |  |  |  |  |
|  |  |  |  |  |
|  |  |  |  |  |
|  |  |  |  |  |

| 8. **Physical Activity** | | | | |
| --- | --- | --- | --- | --- |
|  | | | | |
| **Question** | | **Response** | | **Code** |
| **Work** | | | | |
| 8.1 | Does your work involve vigorous-intensity activity that causes large increases in breathing or heart rate like *[carrying or lifting* *heavy loads, digging or construction work]*  for at least 10 minutes continuously? | Yes | 1 | *P1* |
|  |  | No | 2  *If No, go to P 4* |  |
| 8.2 | In a typical week, on how many days do you do vigorous-intensity activities as part of your work? | Number of days | └─┘ | *P2* |
| 8.3 | How much time do you spend doing vigorous-intensity activities at work on a typical day? | Hours : minutes | └─┴─┘: └─┴─┘  hrs mins | P3 (a-b) |
| 8.4 | Does your work involve moderate-intensity activity, that causes small increases in breathing or heart rate such as brisk walking *[or carrying light loads]* for at least 10 minutes continuously? | Yes | 1 | P4 |
|  |  | No | 2 *If No, go to P 7* |  |
| 8.5 | In a typical week, on how many days do you do moderate-intensity activities as part of your work? | Number of days | └─┘ | P5 |
| 8.6 | How much time do you spend doing moderate-intensity activities at work on a typical day? | Hours : minutes | └─┴─┘: └─┴─┘  hrs mins | P6 (a-b) |
| **Travel to and from places** | | | | |
| 8.7 | Do you walk or use a bicycle *(pedal cycle)* for at least 10 minutes continuously to get to and from places? | Yes | 1 | P7 |
|  |  | No | 2  *If No, go to P 10* |  |
| 8.8 | In a typical week, on how many days do you walk or bicycle for at least 10 minutes continuously to get to and from places? | Number of days | └─┘ | P8 |
| 8.9 | How much time do you spend walking or bicycling for travel on a typical day? | Hours : minutes | └─┴─┘: └─┴─┘  hrs mins | P9 (a-b) |

| **Recreational activities** | | | | |
| --- | --- | --- | --- | --- |
| The next questions exclude the work and transport activities that you have already mentioned.  Now I would like to ask you about sports, fitness and recreational activities (leisure), | | | | |
| 8.10 | Do you do any vigorous-intensity sports, fitness or recreational *(leisure)* activities that cause large increases in breathing or heart rate like *[running or football]*  for at least 10 minutes continuously? | Yes | 1 | P10 |
|  |  | No  No | 2  *If No, go to P 13* |  |
| 8.11 | In a typical week, on how many days do you do vigorous-intensity sports, fitness or recreational *(leisure)* activities? | Number of days | └─┘ | P11 |
| 8.12 | How much time do you spend doing vigorous-intensity sports, fitness or recreational activities on a typical day? | Hours : minutes | └─┴─┘: └─┴─┘  hrs mins | P12  (a-b) |
| 8.13 | Do you do any moderate-intensity sports, fitness or recreational *(leisure)* activities that cause a small increase in breathing or heart rate such as brisk walking*, [cycling, swimming, volleyball]* for at least 10 minutes continuously?*)* | Yes | 1 | P13 |
|  |  | No | 2  *If No, go to P16* |  |
| 8.14 | In a typical week, on how many days do you do moderate-intensity sports, fitness or recreational *(leisure)* activities? | Number of days | └─┘ | P14 |
| 8.15 | How much time do you spend doing moderate-intensity sports, fitness or recreational *(leisure)* activities on a typical day? | Hours : minutes | └─┴─┘: └─┴─┘  hrs mins | P15 (a-b) |

| **Sedentary behaviour** | | | | |
| --- | --- | --- | --- | --- |
| The following question is about sitting or reclining at work, at home, getting to and from places, or with friends including time spent sitting at a desk, sitting with friends, traveling in car, bus, train, reading, playing cards or watching television, but do not include time spent sleeping.  *[INSERT EXAMPLES] (USE SHOWCARD)* | | | | |
| 8.16 | How much time do you usually spend sitting or reclining on a typical day? | Hours : minutes | └─┴─┘: └─┴─┘  hrs mins | P16  (a-b) |
