# Supplementary material for: Vitamin D Supplementation in Overweight/obese Asian Indian Women with Prediabetes Reduces Glycemic Measures and Truncal Subcutaneous Fat: A 78 Weeks Randomized Placebo-Controlled Trial (PREVENT-WIN Trial)
Source: Sci Rep. 2020 Jan 14;10:220. doi: 10.1038/s41598-019-56904-y (PMC6959323; doi:10.1038/s41598-019-56904-y)
Supplement: Supplementary file 2 — Supplementary Information 2 [file 41598_2019_56904_MOESM2_ESM.docx]

Title: Vitamin D Supplementation in Overweight/obese Asian Indian Women with Prediabetes Reduces Glycemic Measures and Truncal Subcutaneous Fat: A 18-month Randomized Controlled Trial (*PREVENT-WIN* Trial).

Authors; Surya Prakash Bhatt^1,2,3,5^, Ph.D.; Anoop Misra^1,2,5^, MD; Ravindra Mohan Pandey^4^,Ph.D; Ashish Datt Upadhyay^,4^, Ph.D.; Seema Gulati^1,2^, PhD; Namrata Singh^1,2^, MSc

^1^Diabetes Foundation (India), Safdarjung Development Area, New Delhi 110016, India.

^2^National Diabetes Obesity and Cholesterol Foundation (N-DOC), Safdarjung Development Area, New Delhi 110016, India.

^3^Department of Pulmonary Medicine and Sleep Disorders, and ^4^Biostatistics, All India Institute of Medical Sciences, Ansari Nagar, New Delhi 110029, India

^5^Fortis C-DOC Center of Excellence for Diabetes, Metabolic Diseases, and Endocrinology, B-16, Chirag Enclave, New Delhi, India

Address for correspondence and reprint request to:

Professor Anoop Misra

Chairman, Fortis-CDOC Center of Excellence for Diabetes, Metabolic Diseases and Endocrinology, B-16, Chirag Enclave, New Delhi, India.

Ph: 91-11-4277-6222 (Ext: 5030); Fax: 91-11-4277-6221; Email: [anoopmisra@gmail.com](mailto:anoopmisra@gmail.com)

**Supplement 2: Table 1: Pulse wave velocity according to hypertensive and non hypertensive individuals.**

| Variables |  | Week 0 | | Week 52 | | Week 78 | | Overall p value  (Between the groups) | Overall Effect Size (95%CI)  Using GEE Method |
| --- | --- | --- | --- | --- | --- | --- | --- | --- | --- |
|  |  | Intervention (n, 17) | Placebo  (n, 27) | Intervention (n, 17) | Placebo  (n, 27) | Intervention (n, 17) | Placebo  (n, 27) |  |  |
| Pulse wave velocity (m/s) | Hypertensive | 8.2±2.1 | 6.96±1.65 | 8.20±2.21 | 6.53±2.44 | 8.20±2.21 | 6.53±2.4 | 0.08 | -1.34 (-2.7, 0.04) |
|  | Non-hypertensive | 6.85±2.0 | 7.20±1.6 | 6.8±2.0 | 7.01±1.5 | 6.8±2.0 | 7.04±1.5 | 0.57 | 0.28 (-71, 1.28) |
| ASP (mmHg) | Hypertensive | 113.6±7.0 | 111.3±4.5 | 113.1±7.5 | 111.4±4.5 | 113±7.5 | 111.6±3.5 | 0.40 | -1.5 (-5.3, 2.4) |
|  | Non-hypertensive | 111.6±3.9 | 112.5±2.0 | 111±3.28 | 112.6±2.0 | 111.04±3.3 | 112.5±2.06 | 0.13 | 1.42 (-0.42, 3.2) |
| APP (mmHg) | Hypertensive | 31±5.5 | 31±3.3 | 31.4±5.5 | 31.9±4.7 | 31.5±5.5 | 31.9±4.7 | 0.85 | 1.58 (-0.12, 3.3) |
|  | Non-hypertensive | 30.04±3.3 | 31.7±3.6 | 30.04±3.8 | 31.5±2.1 | 30.04±3.4 | 31.5±2.1 | 0.06 | 3.19 (-0.18, 8.27) |
| Alx@HR75 (%) | Hypertensive | 26.4±9.2 | 28.9±5.0 | 26.6±8.7 | 28.2±5.7 | 26.6±8.7 | 28.3±8.2 | 0.42 | 2.16 (-3.1, 7.5) |
|  | Non-hypertensive | 26.9±9.0 | 29±10.4 | 27.4±9.0 | 30.8±7.9 | 27.4±9.0 | 30.9±7.9 | 0.21 | 3.19 (-0.18, 8.27) |
| Ejection duration (%) | Hypertensive | 39.4±4.8 | 39.7±4.4 | 39.3±4.6 | 40.1±3.8 | 39.3±4.6 | 40.2±3.8 | 0.61 | 0.73 (-2.0, 3.5) |
|  | Non-hypertensive | 39.5±3.9 | 38.7±2.8 | 39.6±3.8 | 42.6±17.1 | 39.6±3.9 | 42.6±17.1 | 0.45 | 2.6 (-4.3, 9.5) |
| SEVR (%) | Hypertensive | 135.6±24.3 | 134.2±25 | 136±23.6 | 130.3±22.8 | 136.0±23.6 | 130.3±22.1 | 0.60 | -3.9 (-18.8, 11.0) |
|  | Non-hypertensive | 136.2±23.1 | 138.2±17 | 135±22.9 | 138.2±26.1 | 135.6±22.9 | 138.2±26.1 | 0.67 | 2.8 (-10.2, 15.8) |

Results are shown as mean± SD. p value <0.05 statistically significant. Intention-to-treat analyses has been conducted. ASP, aortic systolic pressure (mm Hg); APP, aortic pulse pressure (mm Hg) , Alx@75 % HR, augmentation index @75% HR, SEVR, subendocardial viability ratio; GEE, generalized estimating equation.
